# Low-cost, Low-bias and Low-input RNA-seq with High Experimental Verifiability based on Semiconductor Sequencing

**DOI:** 10.1038/s41598-017-01165-w

**Published:** 2017-04-21

**Authors:** Zhibiao Mai, Chuanle Xiao, Jingjie Jin, Gong Zhang

**Affiliations:** Key Laboratory of Functional Protein Research of Guangdong Higher Education Institutes, Institute of Life and Health Engineering, Jinan University, Guangzhou, 510632 China

## Abstract

Low-input RNA-seq is powerful to represent the gene expression profiles with limited number of cells, especially when single-cell variations are not the aim. However, pre-amplification-based and molecule index-based library construction methods boost bias or require higher throughput. Here we demonstrate a simple, low-cost, low-bias and low-input RNA-seq with ion torrent semiconductor sequencing (LIEA RNA-seq). We also developed highly accurate and error-tolerant spliced mapping algorithm FANSe2splice to accurately map the single-ended reads to the reference genome with better experimental verifiability than the previous spliced mappers. Combining the experimental and computational advancements, our solution is comparable with the bulk mRNA-seq in quantification, reliably detects splice junctions and minimizes the bias with much less mappable reads.

## Introduction

With the increasing precision of biological research, low-input RNA-seq have been shown its power in revealing gene expression of limited number of cells. RNA-seq of 50~400 cells yielded an *R* ^2^ > 0.9 against bulk RNA-seq, thus can represent the population^1, 2^, which is sufficient and cost-efficient in the studies where single-cell variations are not the aim. However, the low input of less than 100 pg polyA + mRNA (equivalent to ~1000 human cells) is difficult for library construction using any conventional library construction methods. To tackle this hindrance, intensive amplification was necessary in previous approaches^3^. However, the sequence-specific bias is also exponentially amplified, resulting in a loss of linearity to the bulk RNA-seq^4^. A useful idea to compensate the bias is to label each transcript with random tags^5, 6^. Identical tag refers to the same transcript, thus eliminates the amplification bias. However, the sequencing throughput needs to be elevated to adequately sequence the fragments with low amplification efficiency, and the tag can be added only to the 3′-end^6^ or 5′-end^5^, thus is unable to cover the entire transcript. Using poly-dT to reverse transcribe polyA + mRNA is possible to generate full-length cDNA^4^. However, it cannot be applied to polyA- non-coding RNA analysis, and the 3′-end bias cannot be eliminated. Therefore, a simple and low-bias method for such low-input RNA-seq is highly appreciated in the community.

In another aspect, the experimental validity of the RNA-seq results is still to be improved: the tiny amount of input RNA is consumed in the sequencing experiment, leaving little chance for experimental validation. This necessitates extremely accurate and robust algorithms to minimize the false positives and false negatives. It has been shown that different aligners performed dramatically different in experimental verifiability^7–9^, especially in detecting splice variants: the junction detection sensitivity of the mainstream splice mappers can be as low as 65.36%^9^.

To systematically solve these two problems, we developed a low-cost, low-bias low-input RNA-seq process with high experimental verifiability combining experimental and computational approaches.

## Results

### Reducing bias using Low-Input, Equal-Amplification (LIEA) method

**Figure 1:**
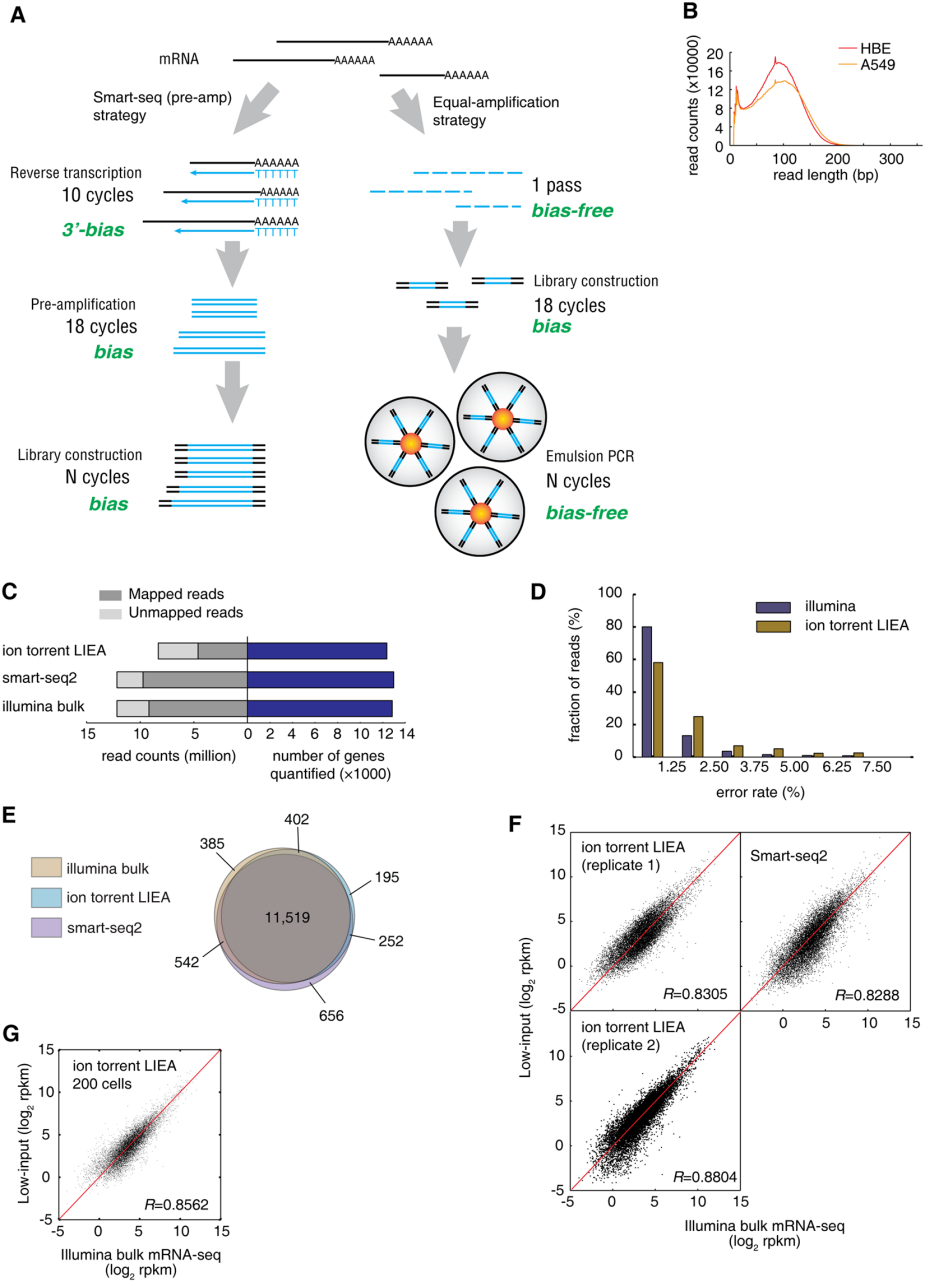
Low-input and low-bias ion torrent mRNA-seq. (**A**) Principle of the ion-torrent LIEA method and comparison to the smart-seq (pre-amplification) strategy. (**B**) Read length distribution of ion-torrent low-input mRNA-seq. (**C**) Comparison of the total read count, mapped read count and number of genes quantified by ion torrent LIEA method, smart-seq2 and Illumina bulk RNA-seq, for HBE cell line. (**D**) Error rate distribution of two platforms. An error is defined as a mismatch or indel nucleotide when aligned to the reference sequence. (**E**) Overlap of quantified genes of the three methods. (**F**) Comparison of the gene expression quantification using two low-input methods against Illumina bulk mRNA-seq, all for HBE cells. Ion torrent LIEA method was carried out twice as biological replicates. (**G**) Comparison of the gene expression quantification using LIEA method with 200 cells as starting material and Illumina bulk mRNA-seq.

The Equal-Amplification strategy is designed to minimize 3′-bias and sequence-specific amplification bias. To avoid 3′-bias, the input polyA + mRNA was randomly fragmented, and the adapters were ligated to the fragments. cDNA library was then generated by reverse-transcription. To minimize the amplification of sequence-specific bias, PCR amplification of the cDNA library was performed for limited 18 cycles. Then we took the advantage of the Ion Torrent sequencing technology to amplify each library molecule on separate beads independently in One Touch device. After enough PCR cycles, each library molecule was equally amplified to saturation level (Fig. 1A). Therefore, no bias was introduced in this step, i.e. the total bias would be no greater than high-input protocols. Compared to the index-based molecule counting method, our low-input method should achieve the same linearity with much lower throughput because an easy-to-amplify fragment will result in multiple reads in the molecule counting method.

We directly utilized the Ion OneTouch2 system to perform this bead-based emulsion PCR process as one step in the Ion Torrent sequencing. The positive beads (with the amplified fragments) can be enriched and directly loaded into the sequencing chip. This means that our approach does not create any additional step compared to the conventional bulk mRNA-seq when using semiconductor sequencing.

### LIEA RNA-seq quantitatively reproduced bulk mRNA-seq results

We applied this LIEA strategy on human mRNA samples from ~10 pg polyA + mRNA (equivalent to mRNA in 50~100 cells, diluted from bulk mRNA) of HBE and A549 cell lines, respectively. The sequencing data was compared with the bulk mRNA-seq data^10^ and Smart-seq2 method^11^ with the same starting materials.

The LIEA method generated an average read length with ~87 nt (Fig. 1B), remarkably shorter than the nominated 200 nt read length of the sequencer, due to the extremely low starting material so that the mRNA was over-fragmented. Ion torrent Proton P1 chip yielded 18.3 M and 16.5 M reads for HBE and A549 cells, respectively. However, half of the ion torrent reads contain low quality nucleotides (Phred score <10) in the first 42 nucleotides and thus were excluded from further analysis. For the HBE dataset, 8.32 M high quality reads were subjected to read mapping (Fig. 1C).

The higher error rates of the ion torrent LIEA method challenges the mapping algorithm on the error tolerance and the accuracy. Therefore, we used the experimentally verified and highly accurate FANSe2 algorithm^7^ to map the reads to human RefSeq-RNA reference sequences. 55.3% of the ion torrent LIEA reads were mapped to the transcriptome references (Fig. 1C), much lower than the Illumina datasets (80.0% for Smart-seq2 and 75.5% for bulk mRNA-seq datasets), due to the higher error rates of the ion torrent sequencer (Fig. 1D). Nevertheless, with only half number of the mapped reads, our LIEA RNA-seq approach quantified almost same number of expressed genes as the normal input RNA-seq (Fig. 1C), providing a solid basis for further analyses. More than 90% of quantified genes (11,519 genes) by the three methods overlap (Fig. 1E), indicating the correctness of LIEA RNA-seq method. Indeed, the LIEA RNA-seq approach showed linear correlation to the bulk mRNA-seq throughout the entire dynamic range (*R* = 0.8305~0.8804 and 0.8237, respectively, Fig. 1F and Supplementary Fig. S1), comparable with the Smart-seq2 method (*R* = 0.8288). In two biological replicates, the ion torrent LIEA RNA-seq method showed both high correlation to the bulk mRNA-seq, showing its reproducibility (Fig. 1F). We further isolated RNA directly from 200 cells of HBE cell line. The total RNA was below the quantification limit of Qubit and Nanodrop, and we estimated the total RNA yield as 198 ± 15 pg using qRT-PCR (Supplementary Fig. S2), among which the mRNA was less than 10 pg. mRNA was blindly extracted from the total RNA and subjected to LIEA method and lead to comparable result to the bulk RNA-seq (*R* = 0.8562, Fig. 1G).

### LIEA RNA-seq minimizes bias

**Figure 2:**
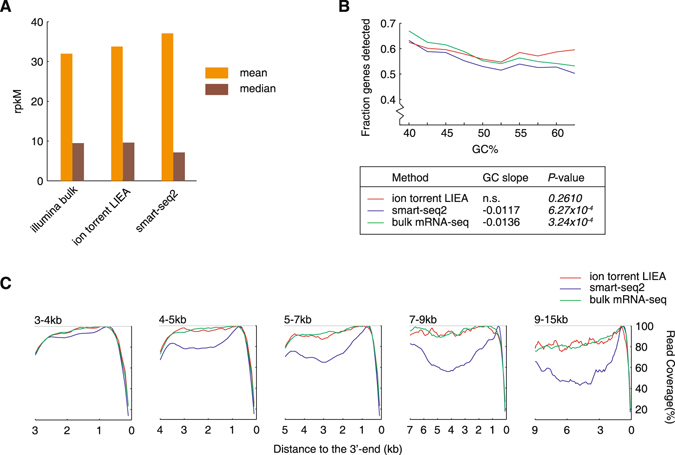
Sequence bias of the low-input methods. (**A**) The mean and median rpkM of quantified genes of the three methods. (**B**) GC bias of the two low-input method and bulk mRNA-seq. Genes with expression level of rpkM > 1 were included in the statistics. (**C**) 3′-end bias of the sequencing reads of the two low-input method and bulk mRNA-seq^4^. Genes were classified according to their lengths. Genes with expression level of rpkM > 1 were included in the statistics.

We next assessed the bias created by the three approaches. Compared with Illumina bulk mRNA-seq, the ion torrent LIEA approach yielded almost identical median rpkM and slightly higher mean rpkM for the quantified genes. In contrast, Smart-seq2 resulted remarkably higher mean rpkM but remarkably lower median rpkM (Fig. 2A). These results indicate that the Smart-seq2 created considerable bias towards high-abundance transcripts, while our LIEA approach performed closer to the bulk mRNA-seq. Our LIEA approach showed almost no GC bias, while Smart-seq2 and bulk mRNA-seq significantly biased towards low GC genes (Fig. 2B). For short mRNAs <4 kb, both our approach and Smart-seq2 could reach 3′-bias-free coverage. For longer mRNAs, Smart-seq2 reads accumulated near the 3′-end of the mRNA, while our approach almost overlapped with the bulk mRNA-seq (Fig. 2C). The low 3′-bias resulted in a uniform read coverage and thus facilitates detection of splice junctions.

### Detecting splice junction using single read by the new algorithm FANSe2splice

**Figure 3:**
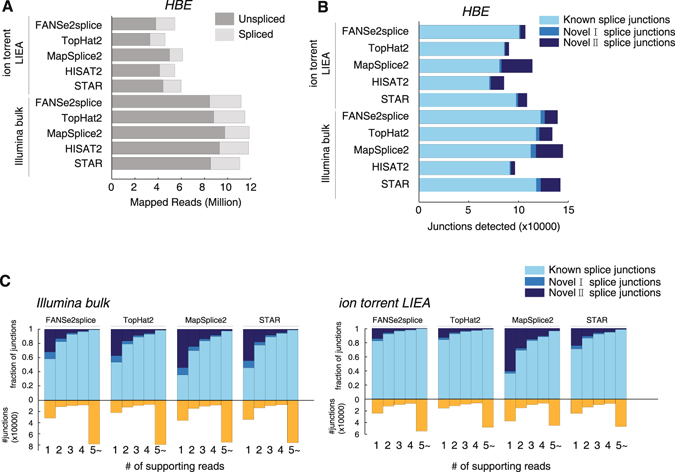
Detecting splice junctions from the ion torrent low-input sequencing dataset using FANSe2splice. (**A**) Comparison of the mapping performance of five algorithms (FANSe2splice, TopHat2, MapSplice2, HISAT2 and STAR) of the HBE mRNA-seq datasets. The mapped spliced reads are indicated as light gray. (**B**) Comparison of the detected junctions by the 5 algorithms. Known splice junctions are the junctions which are already annotated in the database. Novel I splice junctions are unannotated junctions for known genes. Novel II splice junctions are the junctions without any gene annotation. (**C**) The detected splice junctions supported by a number of supporting reads. HISAT2 did not give any read count information in the splice junction result, thus is not compared here.

To reliably detect splice variants from the single-end sequencing data and cope with higher error rate (Fig. 1D) and lower throughput of Ion Torrent sequencers (Fig. 1C), we developed a spliced mapping algorithm FANSe2splice based on our accurate and error-tolerant FANSe2 algorithm. As most other algorithms, FANSe2splice first aligns reads to reference transcriptome and genome sequences in an unspliced way. If the alignment fails, FANSe2splice then aligns the reads as a spliced-read: it allows two hotspots for one read (for details, please see Methods section). We also tested the currently widely-used spliced mappers TopHat2^12^, MapSplice2^13^ HISAT2^14^ and STAR^15^. In general, these algorithms mapped similar number of reads to the reference sequences (Fig. 3A). FANSe2splice identified remarkably more junctions than TopHat2 and HISAT2, while slightly less than MapSplice2 and STAR (Fig. 3B). Notably, the ion torrent low-input method resulted in 88.9% of the detected junctions using much less mappable reads when compared to the Illumina bulk mRNA-seq.

Interestingly, FANSe2splice identified the most known splice junctions among all 5 algorithms (Fig. 3B), which implicated higher robustness of FANSe2splice. We then look deeper into the result, checking the number of supporting reads (i.e. the reads spanning the junction) for each identified splice junction. In both datasets for HBE cells, more than 95% of the junctions with at least 5 supporting reads were previously annotated (Fig. 3C). For Illumina bulk mRNA-seq dataset, FANSe2splice were comparable to TopHat2 with higher number of single-read junctions. For the ion torrent low-input dataset, FANSe2splice detected considerably more junctions with at least 5 supporting reads than the other algorithms, indicating higher robustness in junction detection. MapSplice and STAR detected more than 50% and 30% of novel junctions that are outside of annotated gene regions with only one supporting reads, respectively (Fig. 3C), indicating that these identifications may be suspicious. Moreover, many of the Tophat2/Mapsplice2/HISAT2/STAR solely detected novel junctions are identical to the known junctions in known genes (Supplementary Table S1).

### FANSe2splice detected junctions are experimentally verifiable

It is often difficult to validate the sequencing results for low-input RNA-seq, since all materials are used in the sequencing. Therefore, the robustness and accuracy of the splice junction detection is essential. In this study, we had the chance to validate the splice junctions with bulk mRNA of the two cell lines using RT-PCR. We compared the splice junctions identified by FANSe2splice versus the other three splice mappers, respectively. In all four comparisons, most of the junctions were detected by both algorithms, suggesting correct identifications. The junctions solely detected by one algorithm were subjected to experimental validation. To avoid subjective bias, junctions solely detected by one algorithm were sorted in descending order according to their supportive read counts. We tested the top 5~10 known and novel junctions, which are not identical to any known junctions in known genes. All junctions with less than 5 supportive reads were discarded.

**Figure 4:**
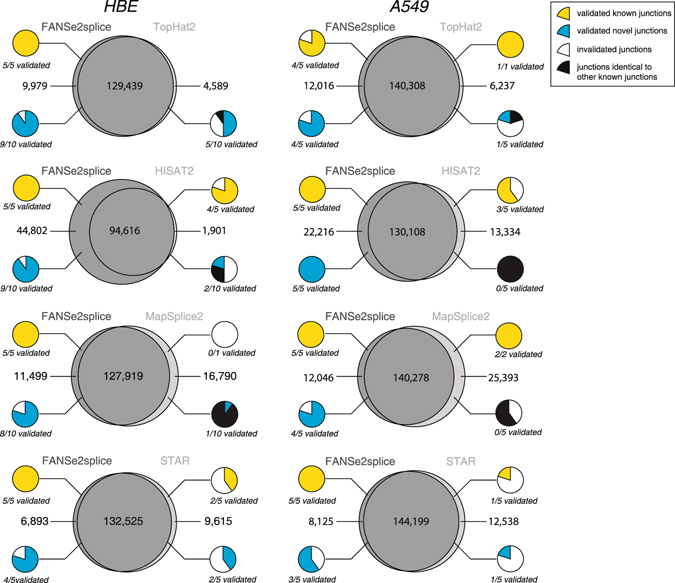
RT-PCR validation of the splice junctions solely detected by one algorithm using bulk mRNA. Comparison of the splice junctions detected by FANSe2splice versus TopHat2, HISAT2, MapSplice2 and STAR, for HBE and A549 cell lines, respectively. The junctions solely detected by one algorithm were tested by RT-PCR. Small pie charts denote the fraction of validated known junctions (yellow) and novel junctions (cyan). The junctions identical to other known junctions were marked as black. Complete experimental evidences for all RT-PCR validations are listed in Supplementary Figs S3–S6. Please see main text for more details.

RT-PCR experiments validated 80%~100% known junctions and 60%~100% of novel junctions solely detected by FANSe2splice, while validated only a small fraction of the junctions solely detected by the other three algorithms, for both cell lines (Fig. 4). Complete experimental evidences for all RT-PCR validations are listed in Supplementary Figs S3~S6.

**Figure 5:**
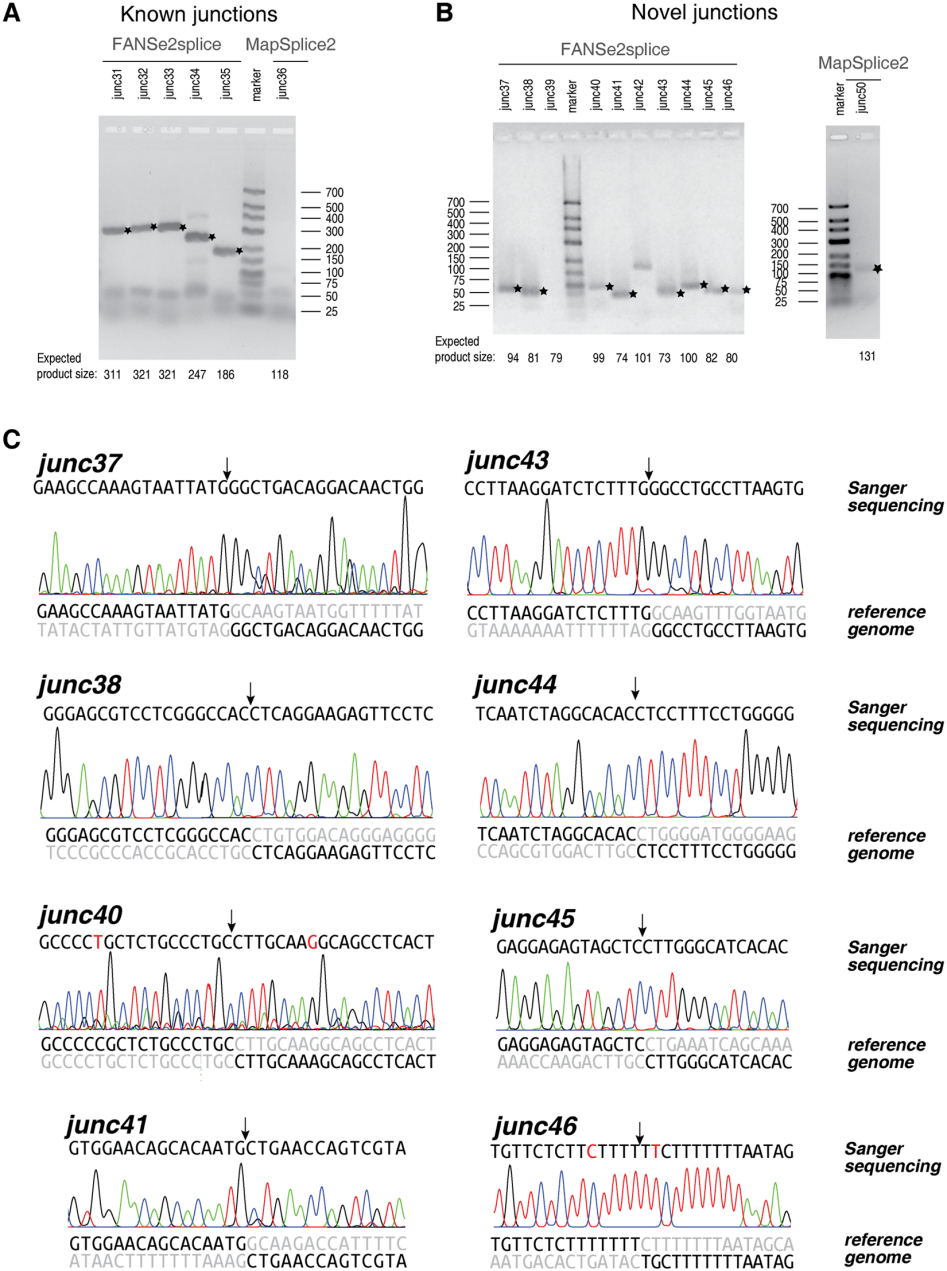
Detailed RT-PCR and Sanger sequencing results of splice junctions solely detected by FANSe2splice versus MapSplice2. (**A**,**B**) RT-PCR validation of the FANSe2splice and MapSplice2 solely detected junctions in HBE, as an example: (**A**) for known junctions and (**B**) for novel junctions. The bands with the expected product sizes were marked with stars. (**C**) Sanger sequencing validation of all 8 novel junctions solely detected by FANSe2splice in (**B**) panel. The arrows indicated the splice site. The exon sequences in the reference genome were shown in black, and the intron sequences were shown in grey. SNPs are marked as red in the sanger sequencing results.

As examples, the RT-PCR results resolved in agarose gel electrophoresis for the junctions solely detected by FANSe2splice versus MapSplice2 are shown in Fig. 5A (known junctions) and Fig. 5B (novel junctions). We further performed Sanger sequencing for all 8 novel junctions solely identified by FANSe2splice in Fig. 5B. All junctions including the junction borders were successfully validated (Fig. 5C), which further consolidated the experimental verifiability of our FANSe2splice algorithm. To be noted, the single-nucleotide polymorphisms (SNPs) in the tested samples (junc40 and junc46) did not confuse the FANSe2splice, even in the case where the SNP is very close to the junction site (junc46), showing its error-tolerance that facilitates the error-prone or SNP-rich sequencing reads. In sum, these results showed that FANSe2splice provides very low false positive rates in detecting splice junctions.

### Ion torrent LIEA RNA-seq facilitates the low-abundance splice junction detection

**Figure 6:**
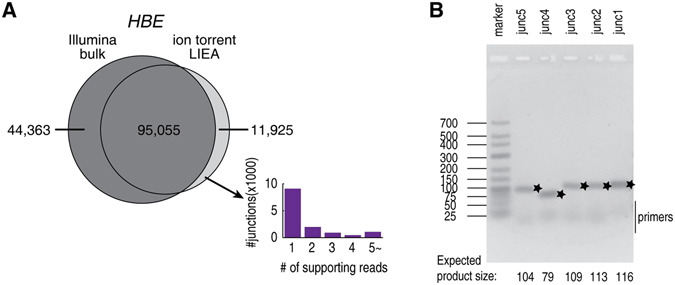
Junctions detected by ion torrent LIEA RNA-seq. (**A**) The Venn diagram of the junctions detected by Illumina bulk mRNA-seq and ion torrent LIEA RNA-seq using the algorithm FANSe2splice. The distribution of the supporting reads of the junctions that solely identified by ion torrent LIEA method. (**B**) RT-PCR validation of mRNAs that were solely detected by ion torrent low-input method, not by the Illumina bulk mRNA-seq. The bands with the expected product sizes were marked with stars. For details, please refer to Supplementary Table S2.

The high accuracy and experimental verifiability of FANSe2splice unleashes the power of low bias and relatively longer read length of ion torrent LIEA RNA-seq. With only half of the mappable reads, the ion torrent LIEA approach identified most of the junctions detected by Illumina bulk mRNA-seq. In addition, ion torrent LIEA method solely identified 11,925 junctions which the Illumina bulk mRNA-seq failed to detect. Over 90% of these splice junctions are lowly expressed junctions (1~4 supporting reads, Fig. 6A). RT-PCR validated all 5 randomly selected such junctions (Fig. 6B and supplementary Table S2). This indicated that ion torrent LIEA RNA-seq leads to lower bias due to the random fragmentation and lack of pre-amplification, and thus provides advantage on detecting lowly expressed splice variants.

## Discussion

It is intriguing that with much lower input material and much lower mappable reads, our ion torrent LIEA strategy showed comparable results to the normal input Illumina dataset on both quantitation and splice junction identification. One major reason is the bias-free emulsion PCR (Fig. 1A) that decreases the read counts of the highly-expressed genes, which are usually more efficiently amplified in library construction. FANSe2splice runs at a reasonable speed even on a normal desktop workstation, in sharp contrast to STAR, which requires huge amount of RAM (Supplementary Fig. S7). To be noted, our accurate and experimental verifiable algorithm compensated the higher error rate and lower throughput. It runs more efficiently than all four other algorithms when processing the error-prone ion torrent reads (Supplementary Fig. S7B). Ion torrent LIEA method does not require additional reagents or customized oligonucleotides and the process can be directly integrated into the standard Ion Torrent sequencing protocols, making such a low-input approach easy to handle and cost-efficient. Moreover, unlike the Smart-seq2 strategy that relies on the poly-dT reverse transcription, our method uses the random fragmentation of the RNA (Fig. 1A), which can be also expanded for low-input polyA- lncRNA sequencing applications. In sum, our strategy provides new possibility to the biological investigations with limited starting materials.

## Materials and Methods

### RNA extraction

Total RNA of HBE and A549 cells were extracted using Trizol reagent as described before^10^. PolyA + mRNA was extracted from total RNA using RNA purification beads (Illumina)^10^.

HBE cells were counted using Millipore Scepter cell counter and diluted to obtain 200 random cells out of the population. To extract RNA from 200 cells, 300 μl Trizol was used with great cautious pipetting to minimize the volume for higher concentration.

### mRNA-seq

For ion torrent low-input method, the library construction was performed using Ion Total RNA-seq Kit v2 (Life Technologies) with minor modifications to the manufacturer’s standard protocol. In brief, mRNA was fragmented using RNase III for 2 min. The fragmented mRNA was blindly processed further without quantification to minimize loss. The cDNA library was amplified for 18 cycles. The entire amplified library was directly and blindly applied to the template preparation using PI Template OT2 200 Kit (Life Technologies) on an Ion OneTouch2 system. The positive template beads were enriched on the Ion OneTouch ES instrument. Each sample was sequenced on Ion Proton semiconductor sequencer using an PI chip. Raw reads were deposited to Gene Expression Omnibus (accession: GSE87660). Reads longer than 42 nt (the length of three seeds in FANSe2splice) with Phred score higher than 10 were kept for analyses.

For Smart-seq2 method, 10pmol anchored oligo-dT primer (5′-AAGCAGTGGTATCAACGCAGAGTACT30VN-3′) and 1 μl dNTP mix were added into the starting mRNA. The mixture was denatured at 72 °C for 3 min and put on ice. First-strand cDNA was synthesized using Superscript II Reverse-Transcription Kit (Invitrogen) including 10 pmol TSO (5′-AAGCAGTGGTATCAACGCAGAGTACrGrG + G-3′). Reverse transcription was performed as 42 °C 90 min, (50 °C 2 min, 42 °C 2 min) for 10 cycles, and 70 °C 15 min. After reverse transcription, 13 μl KAPA HiFi Hotstart Ready Mix (KAPA Biosystems) and 10 pmol ISPCR primer (5′-AAGCAGTGGTATCAACGCAGAGT-3′) were added into the mixture and PCR amplified for 18 cycles. The amplified product was subjected to DNA library construction using NEBNext Ultra DNA Library Prep Kit for Illumina (New England Biolabs). Sequencing was performed on an Illumina HiSeq 4000 sequencer. Reads that passed the Illumina filter were subjected to subsequent analyses. Raw reads were deposited to Gene Expression Omnibus (accession: GSE87660). Since the ion torrent sequencer can only produce single-end reads, the Illumina sequencer was set to single-end mode as well for consistency.

Illumina Bulk mRNA-seq datasets for HBE and A549 cells were done before^10^ (GEO database accession GSE42006).

Human reference genome hg19 (downloaded from UCSC Genome Browser) was used for spliced mapping. NCBI RefSeq-RNA reference sequences (accessed on June 2^nd^, 2014) was used for known RNA annotation. For gene expression quantification, splice variants were merged, and the genes with at least 10 mapped reads were considered as quantified genes^16^.

### Design of FANSe2splice

**Figure 7:**
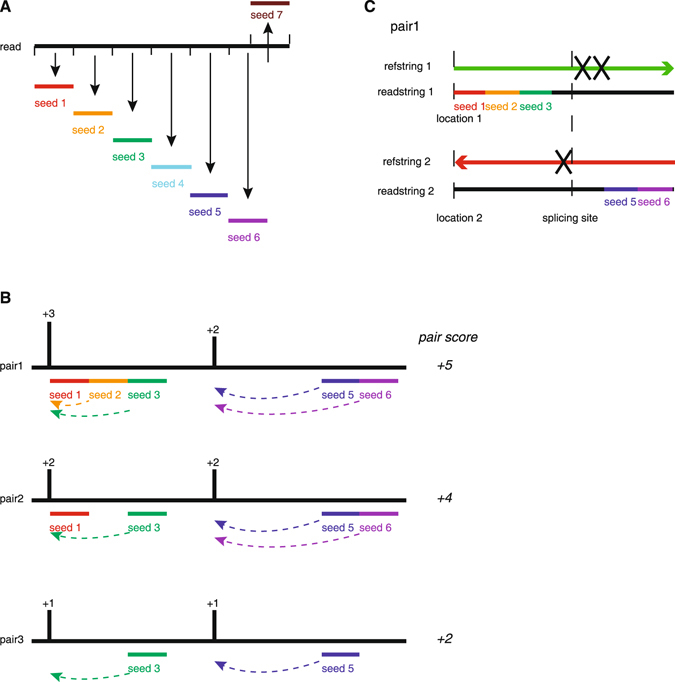
Principle of FANSe2splice algorithm. (**A**) A read is split into consecutive seeds, exactly as the principle in FANSe2. (**B**) Hotspot pairs were generated while seeds were perfectly aligned to the reference genome. The hotspot pair scores are summed up by counting the number of seeds. (**C**) Hotspot pairs are then examined according to the descending order of scores. Examine each hotspot pair to find the best hotspot and determine the position of the splice site.

FANSe2splice is a spliced mapping algorithm based on our accurate and error-tolerant FANSe2 to search for splice junctions. It contains the following steps (Fig. 7):

Step 1. Map reads to the known mRNA reference sequence in an unspliced way using FANSe2 if the genome annotation file is provided. Transcriptome reference sequences are constructed according to the provided gtf/gff file. As the mature mRNA transcripts have already included splice junctions, simple alignment is sufficient to identify the known splice junctions.

Step 2. The unmapped reads are then aligned to the genome reference sequence in an unspliced way by FANSe2. If the genome annotation file is not provided, the task will start at this step to map all reads to the genome reference sequence.

Step 3. The remaining reads from step 2 are considered to contain novel splice junctions. FANSe2Splice splits read into non-overlapping seeds (except the last one in case the read length cannot be divided evenly by seed length) to make full use of the entire read. This design is similar to that in FANSe2^7^ (Fig. 7A).

Step 4. Find the exact matches of the seed in the reference genome and merge the adjacent matches into hotspots. One read is assumed to generate severe paired hotspots. All possible combinations of hotspot pairs within a given range (set by user) are identified. FANSe2Splice then calculates the scores of the hotspot pairs according to the sum of the hotspot scores (Fig. 7B).

Step 5. The hotspot pairs with higher scores are examined in priority. The unmapped seed, in which the splice junction is assumed to be existed, is aligned carefully from two directions to determine the best position of the splice site (Fig. 7C). This search is also guided by the detection of known junction signals (GT-AG, GC-AG, and AT-AC). Only uniquely mapped reads here were used to identify splice junctions.

FANSe2Splice can be freely downloaded at http://bioinformatics.jnu.edu.cn/software/fanse2splice.

**Parameter settings for mapping algorithms**

For mRNA quantification (using the algorithm FANSe2):

Illumina datasets: -E2 -S14 -I1 -M1 -B1 -U0 -Y1 -A0

Ion torrent Proton datasets: -E3 -S14 -I1 -M1 -B1 -U0 -Y1 -A0

For splice junction detection (using GTF annotation file):

FANSe2splice: -E 5 -N 50 -X 500000

TopHat2: -i 50 -I 500000

MapSplice: -i 50 -I 500000

HISAT: –min-intronlen 50 –max-intronlen 500000

STAR: –outFilterType BySJout –outFilterIntronMotifs RemoveNoncanonical –outFilterMultimapNmax 1 –alignIntronMin 50 –alignIntronMax 500000

Software version:

TopHat2: v2.0.13

MapSplice: v2.1.8

HISAT: 2.0.0-beta

STAR: 2.5.2b

### RT-PCR validation of mapping results for RNA-seq

The splice results were compared from FANSe2splice to TopHat2, MapSplice2, HISAT2 and STAR, then the lists of splice junctions that one program can find while the other one cannot will be generated. There are eight lists, two lists are junction splices that were solely identified by FANSe2Splice or TopHat2, two lists are junction splices that were solely identified by FANSe2Splice or MapSplice2, two lists are junction splices that were solely identified by FANSe2Splice or HISAT2 and two lists are junction splices that were solely identified by FANSe2Splice or STAR. And those splice results were split into known junctions and novel junctions according to whether the splice junctions had been annotated or not.

In HBE sample, top five known junctions and top ten/top five novel junctions were selected, while in A549 sample, top five known junctions and top five novel junctions were selected. The selected junctions would be next experimental validation.

Total RNA was isolated by TRIzol Reagent (Invitrogen) respectively from HBE and A549, and then was treated by RNase-free DNase I (Fermentas). After extracted by TRIzol Reagent (Invitrogen), total RNA was reverse-transcribed to generate cDNA by RevertAid Premium Reverse Transcriptase (Fermentas).

We perform PCR to validate the lists of junction splices by using splice-specific primers and DreamTaq Green Mix (Fermentas). The splice-specific primers were designed by the online tool NCBI PrimerBLAST (http://www.ncbi.nlm.nih.gov/tools/primer-blast/, Supplementary Tables S1 and S2). The PCR reaction was set as 95 °C denaturation for 30 seconds, 55 °C annealing for 30 seconds, and 72 °C elongation for 30 sec (amplicon size <500 bp) or 1 min (amplicon size < 1000 bp). 38 PCR cycles were performed for each reaction. The PCR products were electrophoresed on the 2.7~3% agarose gels and visualized by SybrGreen staining. If necessary, the PCR products were subjected to capillary Sanger sequencing for validation.

## Electronic supplementary material

Supplementary Materials

Supplementary Table S1
