# Supplementary material for: Low-cost, Low-bias and Low-input RNA-seq with High Experimental Verifiability based on Semiconductor Sequencing
Source: Sci Rep. 2017 Apr 21;7:1053. doi: 10.1038/s41598-017-01165-w (PMC5430657; doi:10.1038/s41598-017-01165-w)
Supplement: Supplementary file 1 — Supplementary Materials [file 41598_2017_1165_MOESM1_ESM.pdf]

Low-cost, Low-bias and Low-input RNA-seq with High Experimental Verifiability based on Semiconductor Sequencing

Zhibiao Mai, Chuanle Xiao, Jingjie Jin, Gong Zhang *

SUPPLEMENTARY MATERIALS

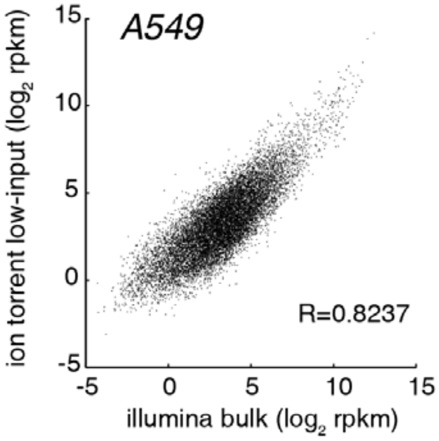

Supplementary Fig. S1: Comparison of the gene expression of A549 mRNA using ion torrent LIEA method and Illumina bulk mRNA-seq. Similar to Fig. 1F.

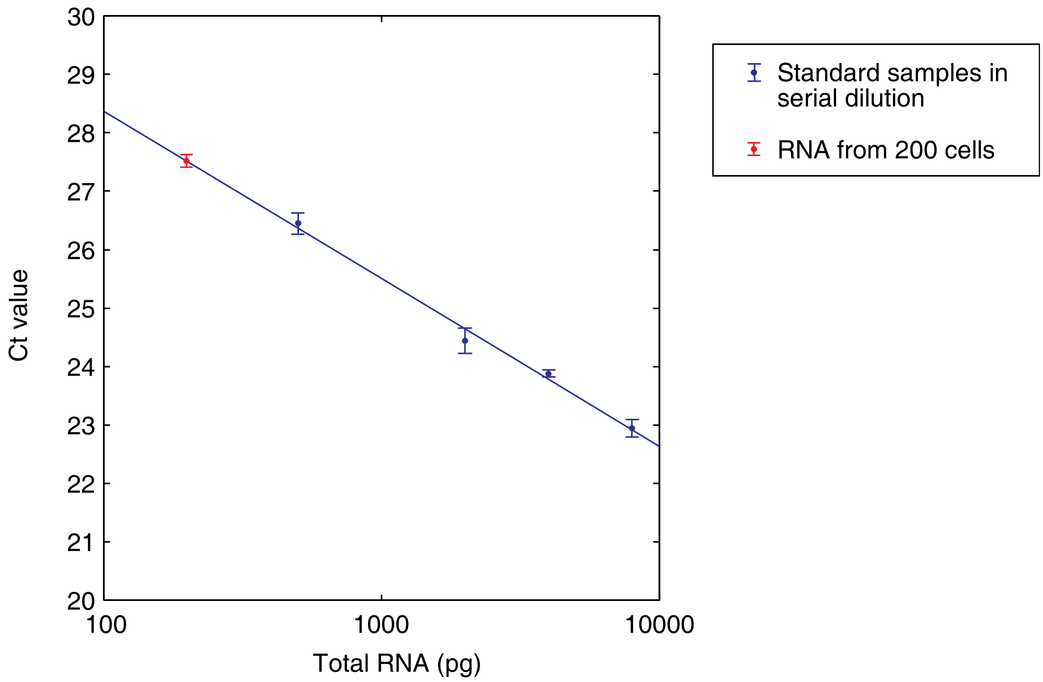

Supplementary Fig. S2: qRT-PCR of RPS3A gene (RefSeq-ID: NM_001006) gene using bulk total RNA and the total RNA isolated from 200 cells. The PCR primers were junc141 (Supplementary Table S1). Bulk total RNA from HBE cells were quantified and serial diluted as standard samples (blue dots, represented as mean ± SD). The standard curve was deduced using linear regression (blue line). The total RNA from 200 HBE cells yielded Ct value of 27.52 ± 0.11 (red dot), corresponding to approximately 198 ± 15 pg.

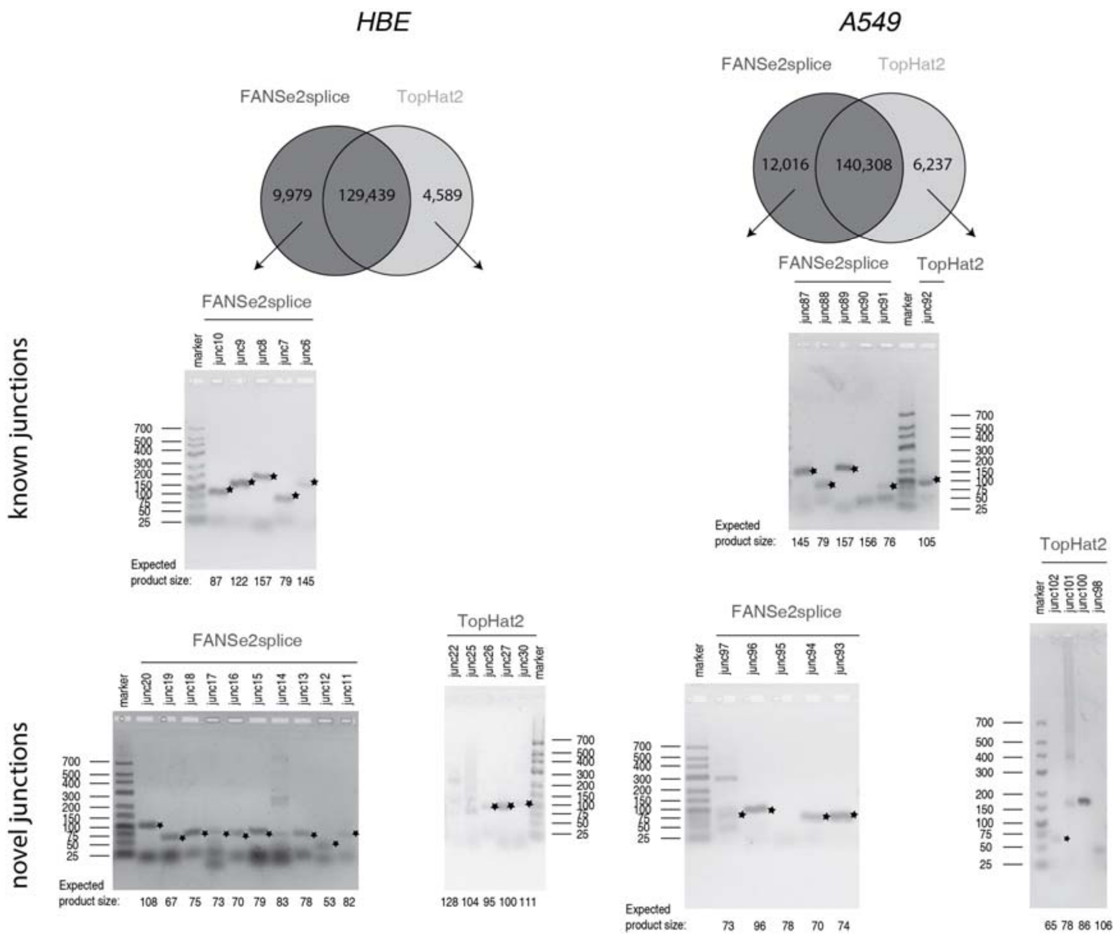

Supplementary Fig. S3: Experimental validation of the splice junctions solely detected by FANSe2splice and TopHat2 in two cell lines, respectively. Validation were performed for the known junctions and novel junctions, respectively, and according to the number of supporting reads in descending order. Detailed junction coordinates, supporting read counts and primer sequences were listed in Supplementary Table S1. The "novel" junctions which are identical to the known junctions in known genes are neglected (see details in Supplementary Table S1).

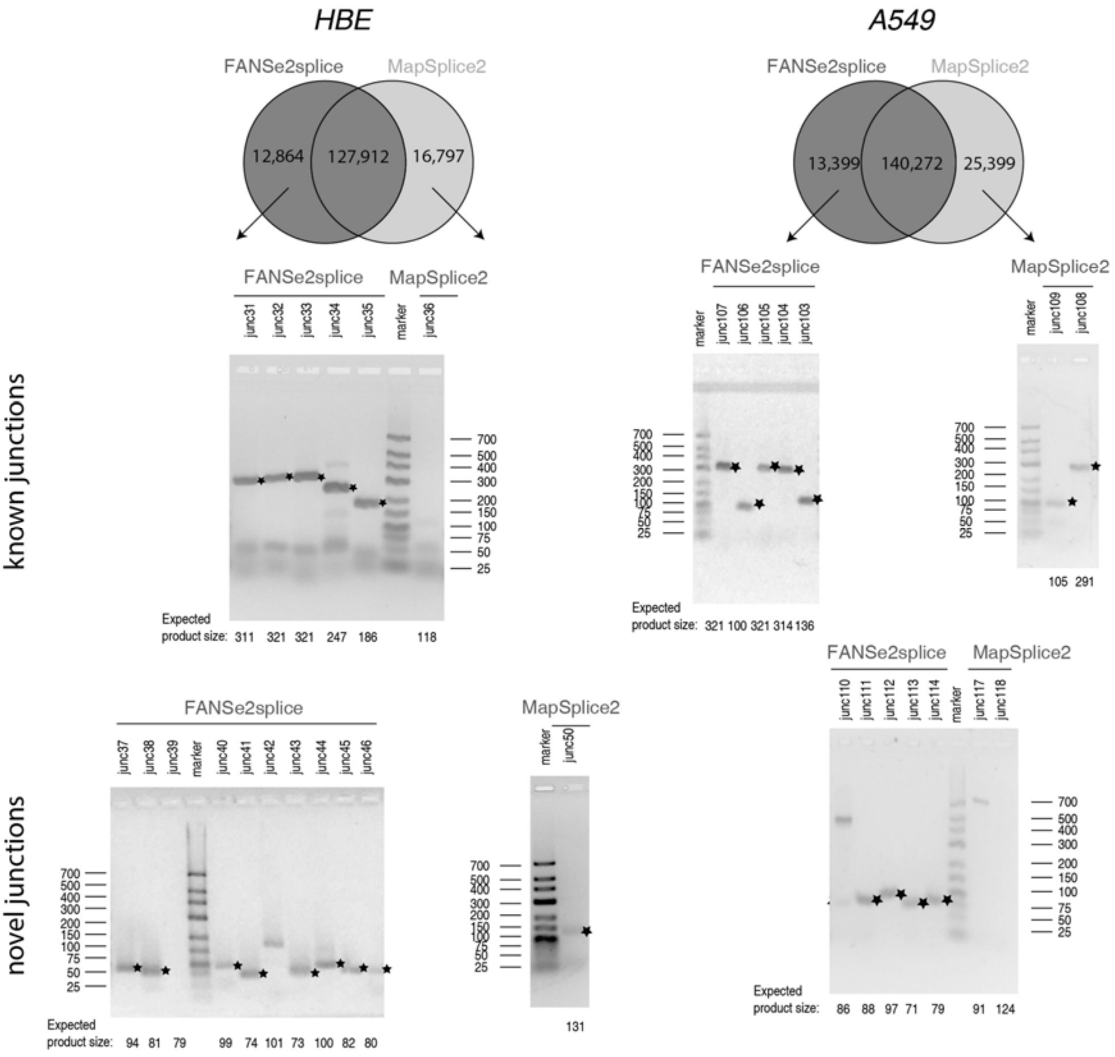

Supplementary Fig. S4: Experimental validation of the splice junctions solely detected by FANSe2splice and MapSplice2 in two cell lines, respectively. Validation were performed for the known junctions and novel junctions, respectively, and according to the number of supporting reads in descending order. Detailed junction coordinates, supporting read counts and primer sequences were listed in Supplementary Table S1. The “novel” junctions which are identical to the known junctions in known genes are neglected (see details in Supplementary Table S1).

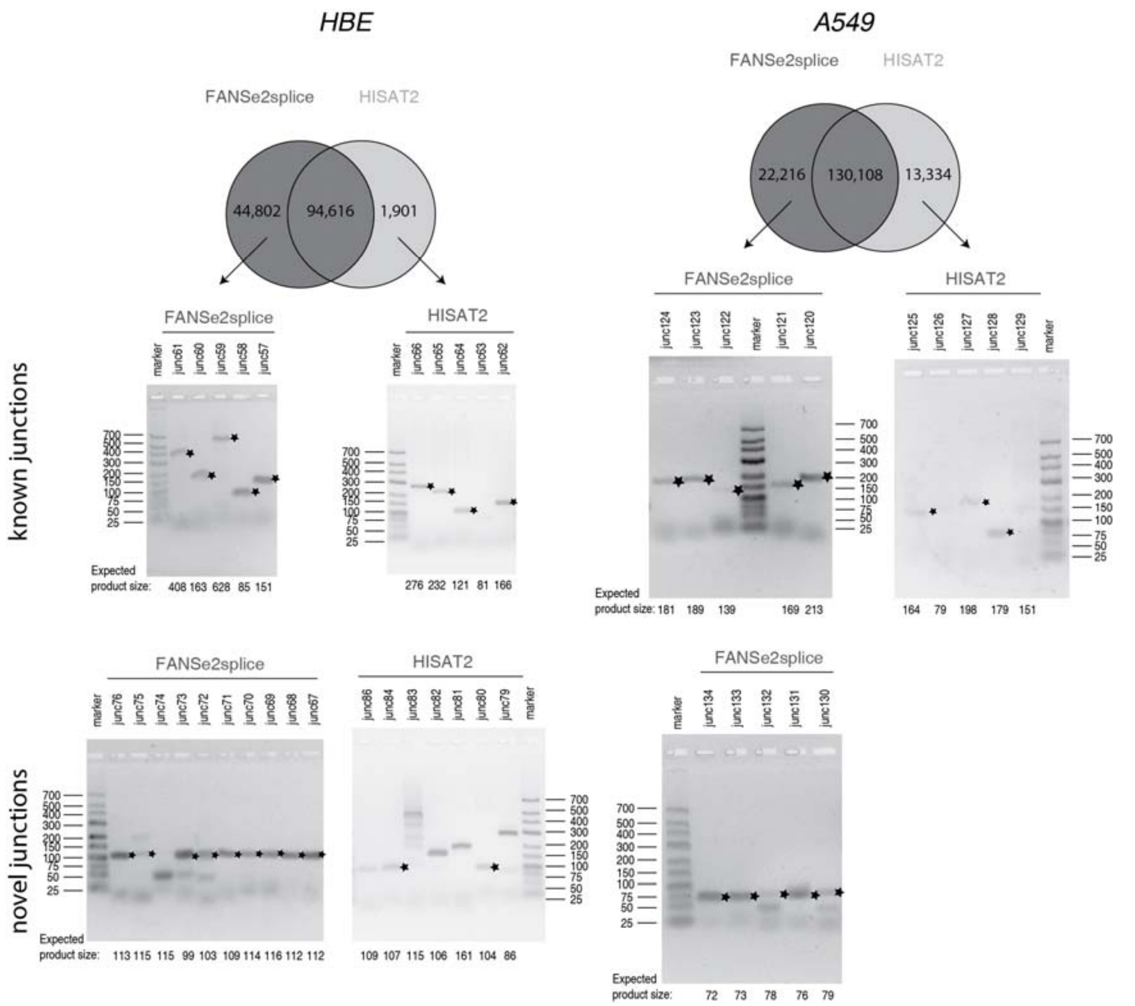

Supplementary Fig. S5: Experimental validation of the splice junctions solely detected by FANSe2splice and HISAT2 in two cell lines, respectively. Validation were performed for the known junctions and novel junctions, respectively, and according to the number of supporting reads in descending order. Detailed junction coordinates, supporting read counts and primer sequences were listed in Supplementary Table S1. The "novel" junctions which are identical to the known junctions in known genes are neglected (see details in Supplementary Table S1).

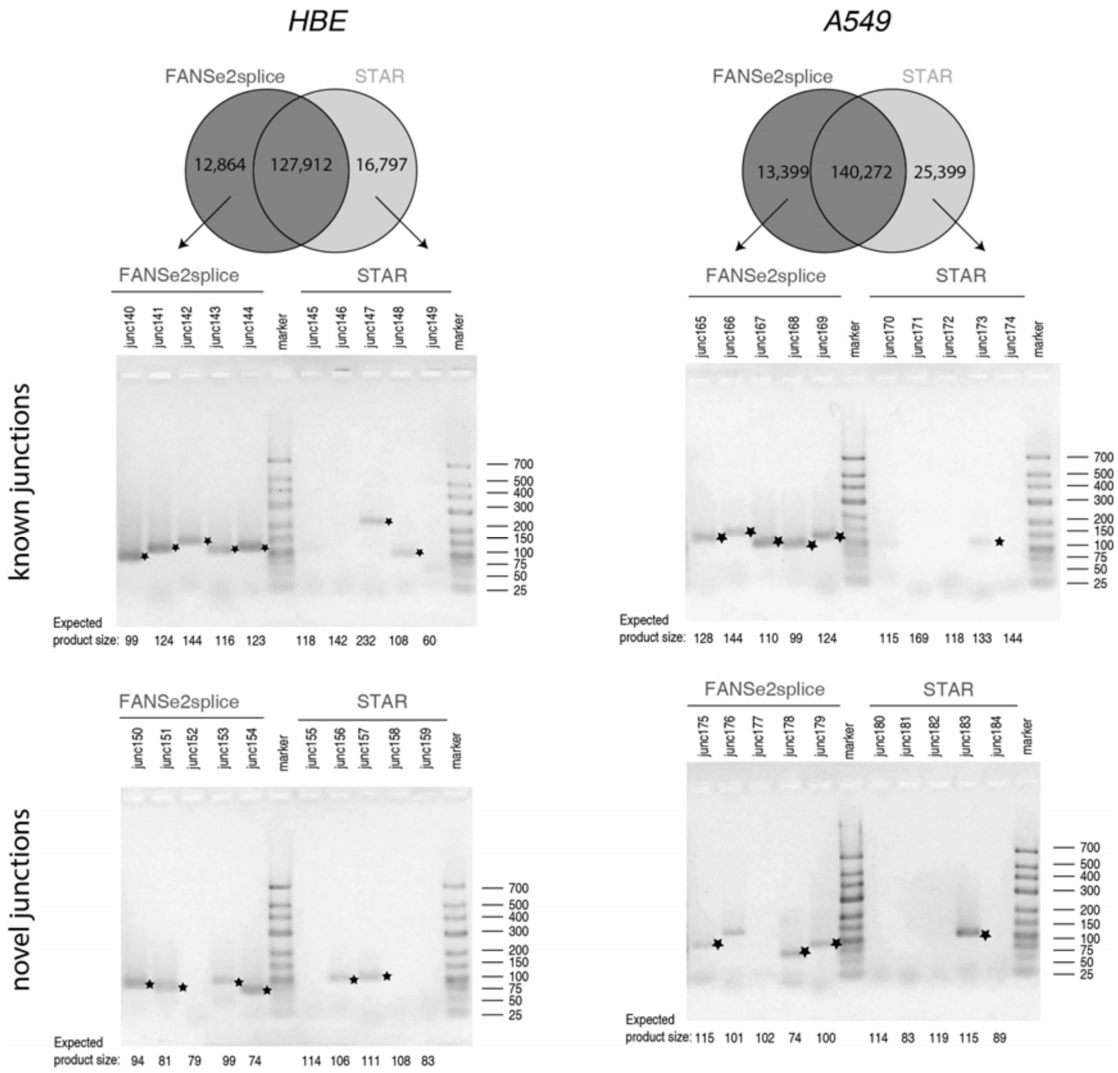

Supplementary Fig. S6: Experimental validation of the splice junctions solely detected by FANSe2splice and STAR in two cell lines, respectively. Validation were performed for the known junctions and novel junctions, respectively, and according to the number of supporting reads in descending order. Detailed junction coordinates, supporting read counts and primer sequences were listed in Supplementary Table S1. The "novel" junctions which are identical to the known junctions in known genes are neglected (see details in Supplementary Table S1).

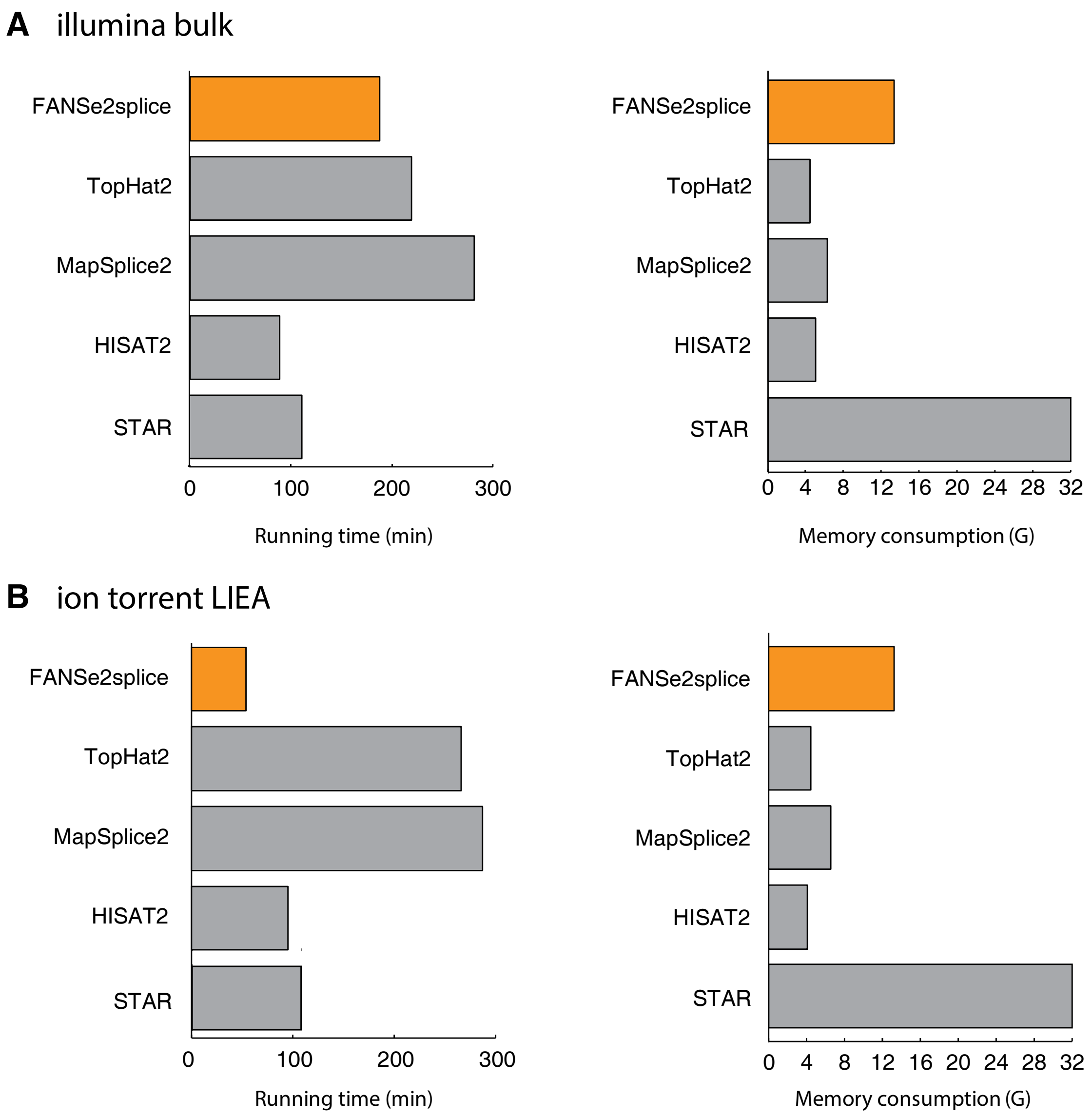

Supplementary Fig. S7: Speed and scalability of the tested algorithms. Running time and memory consumption to process the Illumina bulk mRNA-seq dataset (A) and ion torrent LIEA dataset (B) of the four tested algorithms (except STAR) are recorded on a workstation with dual Intel Xeon X5650 CPUs and 16GB RAM installed. STAR crashed with 16GB and 32GB RAM and ran smoothly with 64GB RAM.

Table S2. RT-PCR validation of randomly selected five splice junctions that are soly identified in HBE mRNA-seq ion torrent dataset (see Fig. 6B).

Junction name	Chromosome	Intron left site	Intron right site	Read count	Validated
junc1	chr12	49522547	49579601	19	Yes
junc2	chr15	72502200	72502690	12	Yes
junc3	chr12	49522357	49579411	9	Yes
junc4	chr20	60962441	60962665	8	Yes
junc5	chr22	24309666	24373704	6	Yes

Junction name	Forward primer (5'-3')	Reverse primer (5'-3')	Expected product size (nt)
junc1	CCTCGCTGCTCATGGAAC	GGATGGAGTTGTAGGGTCAAC	116
junc2	AACTGGGCTCATCAAGGAGC	CCACAGGATGTTCTCGTCAC	113
junc3	CCTGAGATTTGATGGACCCTG	CAGAGATGACAGGGGCATATG	109
junc4	TACGTGCCGCGGAAAGCTC	TCGGCCACGTTCATCTGGAT	79
junc5	CGTGAACGTGACGGTACG	TCGGCGGTGCCCATACG	104
